# Uniformity and Efficacy of Dry Powders Delivered to the Lungs of a Mycobacterial-Surrogate Rat Model of Tuberculosis

**DOI:** 10.1007/s11095-021-03146-1

**Published:** 2021-12-23

**Authors:** Keiji Hirota, Yutaka Hirai, Takehisa Nakajima, Satoru Goto, Kimiko Makino, Hiroshi Terada

**Affiliations:** 1 Faculty of Pharmaceutical Sciences, Tokyo University of Science, 2641 Yamazaki, Noda, Chiba, 278-8510 Japan; 2 Center for Drug Delivery Research, Faculty of Pharmaceutical Sciences, Tokyo University of Science, 2641 Yamazaki, Noda, Chiba, 278-8510 Japan; 3 Center for Physical Pharmaceutics, Research Institute for Science and Technology, Tokyo University of Science, 2641 Yamazaki, Noda, Chiba, 278-8510 Japan; 4 Present Address: Formulation Development Department, Chugai Pharmaceutical Co., Ltd., 5-5-1, Ukima, Kita-ku, Tokyo, 115-8543 Japan; 5 Niigata University of Pharmacy and Applied Life Sciences, 265-1, Higashijima, Akiha-ku, Niigata, 956-8603 Japan

**Keywords:** dry powders, homogeneous distribution, pulmonary administration, tuberculosis, venturi effect

## Abstract

**Purpose:**

Pulmonary administration of dry drug powder is a considered promising strategy in the treatment of various lung diseases such as tuberculosis and is more effective than systemic medication. However, in the pre-clinical study phase, there is a lack of devices for effective delivery of dry powders to the lungs of small rodents. In this study, an administration device which utilizes Venturi effect to deliver dry powders to the lungs homogeneously was developed.

**Methods:**

A Venturi-effect administration device which synchronizes with breathes by use of a ventilator and aerosolizes the dry powders was created. Pulmonary distribution of inhalable dry powders prepared by spray-drying poly(lactic-co-glycolic) acid and an antituberculosis agent rifampicin and anti-tuberculosis effect of the powders on mycobacteria infected rats by administration with the Venturi-effect administration device and a conventional insufflation device were evaluated.

**Results:**

Homogeneous distribution of the dry powders in the lung was achieved by the Venturi-effect administration device due to efficient and recurring aerosolization of loaded dry powders while synchronizing with breathes. Amount of rifampicin delivered to the lungs by the Venturi-effect administration device was three times higher than that by a conventional insufflation device, demonstrating three times greater antimycobacterial activity.

**Conclusions:**

The Venturi-effect administration device aerosolized inhalable antituberculosis dry powders efficiently, achieved uniform pulmonary distribution, and aided the dry powders to exert antituberculosis activity on lung-residing mycobacteria.

## Introduction

Inhalation of therapeutic agents is expected to be effective in the treatment of pulmonary diseases. It has been reported that microspheres with an aerodynamic diameter of 1–5 μm reach and deposit in the periphery of the lung efficiently, exerting optimum therapeutic activity (1) against lung diseases such as tuberculosis caused by *Mycobacterium tuberculosis* (MTB) (2–4). It is inferred that the primary target of MTB is the alveolar macrophages that uptake MTB through phagocytosis; additionally, MTB efficiently proliferates in macrophages by using them as an incubator (5). Hence, pulmonary tuberculosis could be effectively treated via the pulmonary administration of antituberculosis agents encapsulated in microparticles, which are taken up by macrophages through phagocytosis.

To administer inhalable microparticles to laboratory animals, nose-only inhalation chambers have been used as they breathe through the nose (6–8). However, these instruments require a large amount of dry powders, and the exact amount of the drug inhaled cannot be determined (9). Furthermore, dry particles are entrapped in unintended locations, such as the nasal cavity, larynx, and gastrointestinal tract (10, 11). It is noteworthy that small rodents do not perform deep breathing, which is required for homogeneous distribution of a drug in the lung. Thus, nose-only inhalation chambers are not practical for conducting efficacy tests of local acting and expensive pharmaceutical formulations.

In contrast, intratracheal insufflation has several advantages: a small amount of dry powder (less than several milligrams) can be aerosolized and deposition at the oropharynx can be avoided. In addition, the dose delivered to the lungs can be quantitatively determined. Intratracheal insufflation has been employed in pulmonary delivery studies to evaluate drug action in small rodents (12–16). However, this method results in inefficient delivery of dry powders to the lung alveoli due to a manual bolus shot of the powders without synchronization with breathing cycles (17) and requires specific conditions based on the properties of the dry powders, such as the de-aggregation of particles (18). Therefore, the incorporation of inhalable microspheres with antituberculosis agents seem to be less effective than oral administration of the same agent (17, 19).

We assumed that an intratracheal insufflator driven by a ventilator would provide complete synchronization with breathing and uniform delivery of dry powders into the lung. In order to confirm the assumption, we developed a novel dry powder insufflator that can be used with a ventilator and was applicable to rats. We achieved a uniform distribution of dry powders composed of poly(lactic-co-glycolic) acid (PLGA) and the antituberculosis agent rifampicin (RFP) (RFP-PLGA) in the rat lung and thus a potent antimycobacterial effect.

## Materials and Methods

### Materials

Rifampicin (Sigma-Aldrich, St. Louis, MI) and PLGA (Wako Pure Chemical Industry [currently FUJIFILM Wako Pure Chemical Corp.], Osaka, Japan) with a molecular weight of 10,000 and with a monomer composition of lactide and glycolide at a ratio of 75:25 were employed to prepare the inhalable microspheres. RFP-PLGA particles were prepared using the spray-drying method as described previously (20). The content of RFP in RFP-PLGA, determined using a spectrophotometer (UV-2450; Shimadzu, Kyoto, Japan), was approximately 14% (w/w), and the volume median diameter, determined using a laser diffraction equipment (LDSA-3500A; Tohnichi Computer Applications Co., Ltd., Tokyo, Japan), was approximately 2 μm. Other characteristics (dry powder flowability, surface images, etc.) of the RFP-PLGA particles are found in previous studies (20, 21). Additionally, RFP-PLGA containing 0.02% (w/w) coumarin 6 (Sigma-Aldrich) (cRFP-PLGA) was used in the examination of lung distribution of the dry powder.

### Animals

Sprague–Dawley rats (Japan SLC Inc., Shizuoka, Japan) weighing 230–350 g were used. Standard diet and water were provided *ad libitum*. All animal studies were approved by the Institutional Animal Care and Use Committee at Tokyo University of Science (Y13044).

### Venturi-Effect Device

**Fig. 1:**
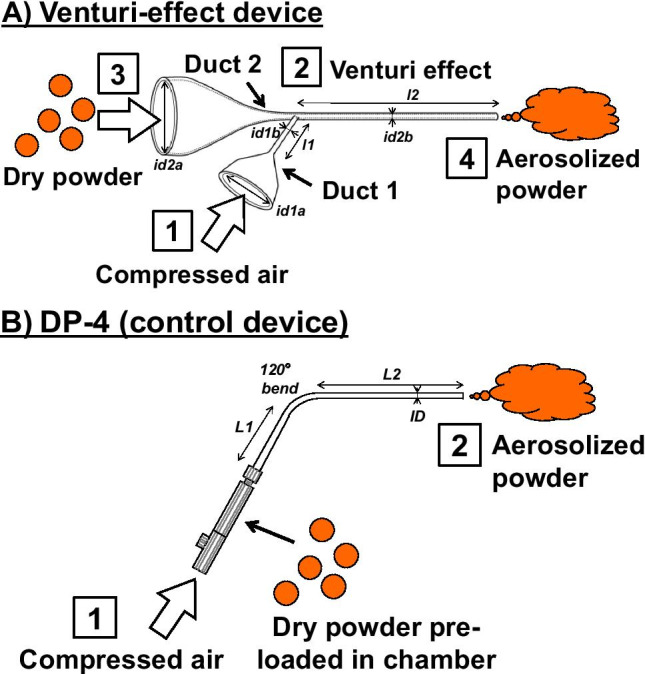
Illustrations of the Venturi-effect device and DP-4 device. Insufflation of dry powders is performed according to the boxed numbers A. Venturi-effect device. When compressed air is supplied through duct 1, the Venturi effect is generated and therefore pressure at the end of duct 1 crossing with duct 2 decreases. The dry powders are then aspirated from duct 2 with an air flow generated by the Venturi effect. Eventually, the dry powders are emitted as a form of aerosol from the tip of the device that is placed in the trachea. Representative dimensions: *l1*, 12 mm; *id1a,* 4.35 mm; *id1b*, 0.30 mm; *l2*, 50 mm; *id2a,* 4.35 mm; *id2b,* 0.83 mm. B. DP-4 device. As a chamber where dry powder is loaded consists of a closed pathway, introduced compressed air directly aerosolizes the powder. Representative dimensions: *L1*, 16 mm; *L2*, 51 mm; *ID,* 0.97 mm.

A Venturi-effect device was designed to aerosolize dry powders and simultaneously introduce aerosols into the lung. The insufflator consisted of a two-way duct: duct 1 for supplying compressed air generated by a ventilator and duct 2 for supplying dry powder (Fig. 1). When compressed air is supplied from duct 1, the static pressure decreases at an intersection between ducts 1 and 2 owing to the increased velocity of the air flow. That is, under the Venturi effect, dry powders were subsequently vacuumed in the insufflator from duct 2 because of the generated negative pressure and were then emitted in the form of aerosolized particles.

### Administration of Dry Powders

Rats anesthetized with an intravenous injection of propofol at 10 mg/kg were laid on their back and the oropharynx was visualized by illuminating the throat over the skin with an optical light source (22). A commercially available insufflator device for rats, such as DP-4 (Penn-Century Inc., Wyndmoor, the company closed in 2015, illustrated in Fig. 1), or the Venturi-effect device developed by us, was then inserted into the trachea up to the carina. Dry powders at a dose of 5 mg were administered as follows. For the DP-4 containing dry powder, 2 mL of compressed air at 50 kPa was provided stepwise seven times; after each step, the insufflator was tapped to emit as much powder as possible. For the Venturi-effect device, compressed air at 50 kPa was supplied from duct 1 for 0.07 s through tubing with inner diameter of 2.5 mm at a frequency of 80 times per minute in order to be synchronized with the breathing of rats and dry powders were administered from duct 2 in a stepwise manner using a vibration feeder during inhalation. As a result, dry powder aerosol was carried by approximately 3 mL of air at each ventilation within 2 min for a dose of 5 mg until the end of administration. In this study, the administration of dry powders was performed using the Venturi-effect device unless otherwise specified.

### Lung Distribution of Dry Powders

After the administration of 5 mg of cRFP-PLGA using the Venturi effect or DP-4 device, under anesthesia with 50 mg/kg pentobarbital *i.p.* injection, the rats were sacrificed by exsanguination through the carotid artery. Thereafter, Ringer’s solution was perfused from the femoral vein to remove erythrocytes, which prevent clear observation of green fluorescence derived from coumarin 6. The lung was then sampled and flattened using glass slides. The distribution of the fluorescent dry powder in the lung was observed using a fluorescent stereotype microscope VB-G05 (Keyence Corp., Osaka, Japan).

To determine the amount of RFP in the lung, five lung lobes, namely, the left and right upper, middle, lower, and accessory lobes, without the trachea and main and lobar bronchi were used. All lobes were minced to approximately 1 mm^3^ in size using a pair of scissors after freeze drying. Acetonitrile was used to extract RFP from the lobes, and the amount of RFP was determined using a spectrophotometer at a wavelength of 475 nm.

### Treatment with RFP-PLGA for Tuberculosis Surrogate Pathogen in the Rat Model

As *M. tuberculosis* is virulent and thus its use for animal study requires specialized facility compatible with Animal Bio Safety Level (ABSL) 3, conducting verification testing of antituberculosis activity is limited. In this study, as a tuberculosis surrogate pathogen, attenuated *Mycobacterium bovis Calmette-Guérin* (BCG) was utilized. The rats were infected with BCG at a cell number of 2 × 10^7^ by intratracheal instillation and then randomly assigned to treatment groups, each consisting of 3–5 rats. After 1 day, 5 mg of RFP-PLGA (approx. 2.8 mg RFP/kg) was administered every day for a total of 7 times or every other day for a total of 4 times per week. The same PLGA microspheres, but without RFP, were also administered at the same dose as the reference. To compare the effect of the device on anti-tuberculosis activity, 5 mg of RFP-PLGA was administered using DP-4 and the Venturi-effect device. As the control for oral administration, RFP (10 mg/kg suspended in 20% (v/v) ethanol) was orally administered daily for a week. In an examination of the combination effect, 5 mg of RFP-PLGA (2.8 mg RFP/kg) together with 1.5 mg (6 mg/kg) of RFP were administered via the pulmonary and oral routes, respectively, once a day for a week. Rats that were infected with BCG without any treatment were referred to as the no treatment group.

### Determination of the Anti-Tuberculosis Activity on Surrogate Pathogen

After the treatments, the rats were sacrificed by exsanguination through the abdominal inferior vena cava under ether anesthesia. The lungs were homogenized with sterile distilled water using a Teflon-glass homogenizer (20 mL, AS ONE, Osaka). The homogenates were serially diluted with distilled water, and aliquots were inoculated on Middlebrook 7H10 agar (Becton, Dickinson and Co., Franklin Lakes, NJ) plates containing 200 U/mL polymyxin B (Wako Pure Chemical Industries, Ltd., Osaka, Japan) and 10 μg/mL amphotericin B (Bristol-Myers Squibb Co., New York, NY). After incubating the plates at 37°C for 3 weeks, colony-forming units (CFUs) were determined from visible colonies on the plates, and the data are expressed as base 10 logarithm.

### Statistical Analysis

Experimental data were statistically analyzed using parametric Student’s *t* test or parametric Bonferroni’s test with the assumption of the same standard deviation as indicated in the figure legends. Statistical analysis was performed using Prism 6 (GraphPad Software Inc., San Diego, CA) and a probability level of 5% (*p* < 0.05) was considered to indicate statistical significance.

## Results

### Generation of Aerosol Using the Venturi-Effect Device

**Fig. 2:**
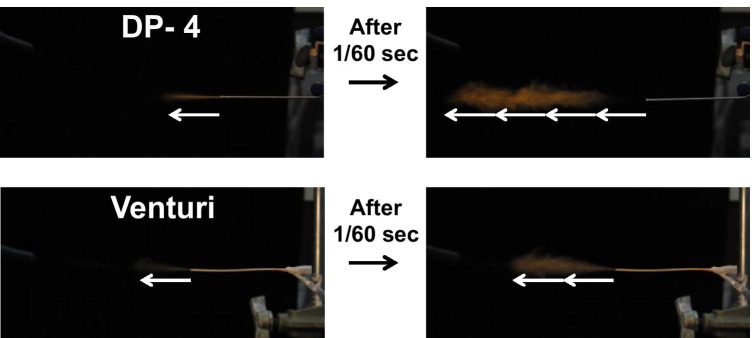
Appearance of the dry powder aerosol generated by DP-4 (top) and the Venturi-effect device (bottom) immediately and at subsequent 1/60 s after dry powder emission (left and right images, respectively). White arrows of the same length indicate travel distance of the generated aerosol. In particular, the aerosol from the Venturi-effect device travelled at half speed of that from DP-4.

Synchronization of breathing rhythms with a bolus shot of dry powder can be one of the key strategies to overcome these problems. Hence, we developed the Venturi-effect device, which is driven by an air supply from a ventilator, as shown in Fig. 1. Open airway duct 2 of the device allowed the dose adjustment of dry powders, and this contributed to repeatable stepwise administration of dry powders. When compressed air was supplied from duct 1 by a ventilator, the dry powder flowed through duct 2 due to the Venturi effect, which generated a negative pressure at the intersection, resulting in the formation of an aerosol insufflated from the tip of the device. As shown in Fig. 2, we compared the mode of insufflation by the Venturi-effect device with that by the one-way duct device DP-4. The aerosol generated by the Venturi-effect device to the open air was more fluent than that by DP-4, although both insufflators were driven at the same air pressure. In addition, as indicated with arrows of the same length in Fig. 2, the flow generated by the Venturi-effect device was relatively slower, approximately half of that generated by DP-4.

### Distribution of Dry Powder in the Lung

**Fig. 3:**
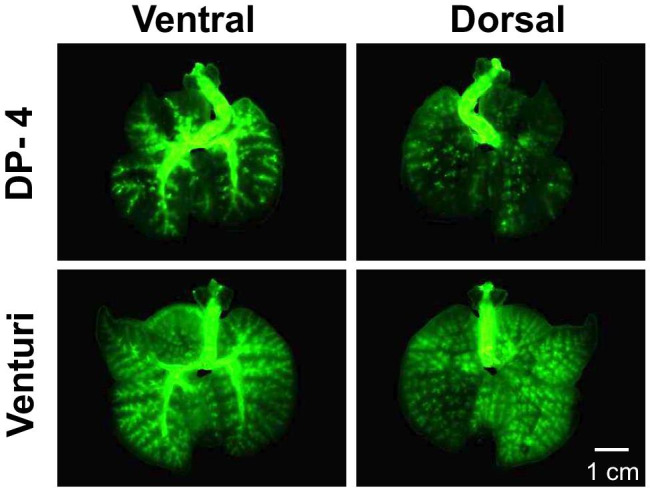
Distribution of cRFP-PLGA insufflated at 5 mg dose in the lung. The images show the lung on the ventral side (left) and dorsal side (right) after insufflation with DP-4 (top) and the Venturi-effect device (bottom).

We then examined the efficiency of the Venturi-effect device to insufflate the dry powder in terms of distribution into the rat lung. As shown in Fig. 3, the lung images were acquired by fluorescent stereomicroscopy after the administration of green-fluorescent cRFP-PLGA by each device. Administration using DP-4 resulted in a poor distribution of the dry powder, and many particles were trapped in the trachea and primary bronchi before entering the lung lobes. In contrast, the Venturi-effect device allowed the dry powder to distribute homogeneously in the lung, although tracheal entrapment was observed. It was interesting to note that the whole lung images from the dorsal side showed fluorescence gathering, resulting in the formation of dots. This image indicates that cRFP-PLGA reached the air sacs located in the deepest area of the lung.

**Fig. 4:**
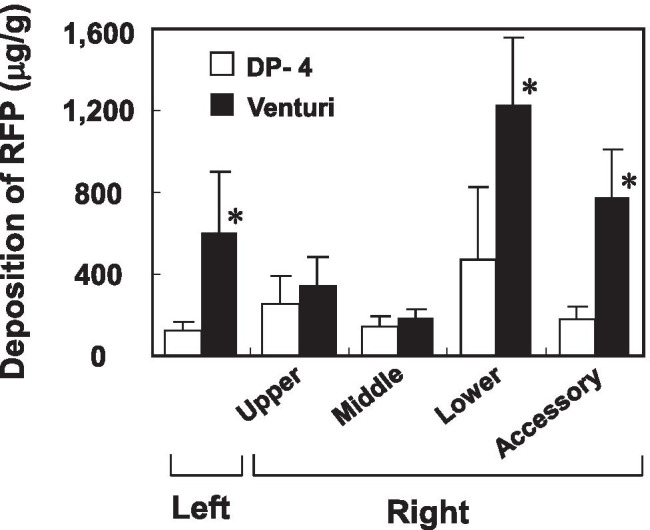
Deposited amount of RFP in the lung lobes. The lung lobes received RFP-PLGA administered by DP-4 or the Venturi-effect device were freeze-dried and subjected to RFP extraction. The values (μg/g) of the deposited amount of RFP per dried weight of the lobes are shown as mean ± S.D. (*n* = 5–6). The asterisk indicates a significant difference at *p* < 0.05 as determined by *t*-test.

**Fig. 5:**
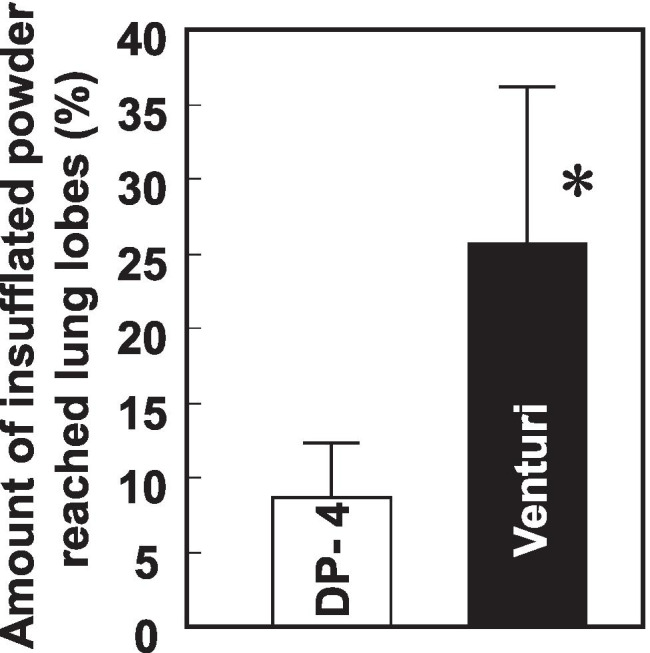
Total amount of the dry powders deposited in the whole lung lobes. The results shown in Fig. 4 are expressed as the relative RFP amount in the whole lung lobes to the total insufflated amount of RFP by both devices. The asterisk indicates a significant difference at *p* < 0.05 as determined by *t*-test.

We then quantified RFP deposited in each lung lobe after insufflation of RFP-PLGA powder using these devices. The Venturi-effect device was able to emit more than 95% of the loaded particles, whereas approximately half of the dry powder remained in the DP-4 device. As shown in Fig. 4, particle delivery by the Venturi-effect device was more efficient than that by DP-4 in all lung lobes. The delivery of RFP in RFP-PLGA by the Venturi-effect device to the left and right accessory lobes was four times higher than that by DP-4. The delivery of RFP to the whole lung by the Venturi-effect device was three times greater than that by DP-4, as shown in Fig. 5.

### Effect of the Venturi-Effect Device on the Antituberculosis Activity of RFP-PLGA

#### Frequency of Dose

**Fig. 6:**
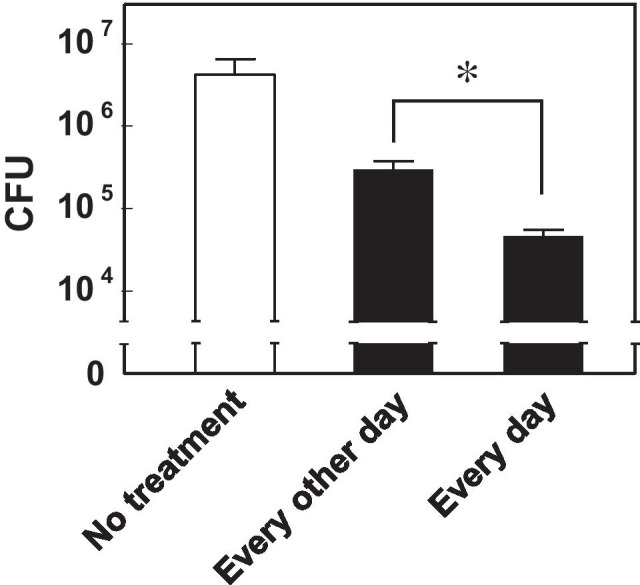
Dose-frequency dependent antituberculosis effect of RFP-PLGA by pulmonary insufflation. RFP-PLGA at 5 mg (2.8 mg RFP/kg) was insufflated by the Venturi-effect device every day (7 times) or every other day (4 times) for 7 days. The data are expressed as mean ± S.D. (*n* = 3). The asterisk indicates statistically significant difference at *p* < 0.05 as determined by *t*-test.

Previously, we reported that sustained release of RFP was achieved with RFP-PLGA 7510 suspended in phosphate-buffered saline (PBS) solution at a neutral pH under 37°C for approximately 4 days, regardless of the presence of a pulmonary surfactant (19). In addition, RFP in the form of RFP-PLGA was taken up by alveolar macrophage and deposited there for 48 h (4). However, when administered RFP-PLGA into the rat lung, a considerable amount of the RFP deposited due to uptake by alveolar macrophage disappeared in 6 h (14). Therefore, we assumed that an interval between pulmonary doses of RFP-PLGA is important for achieving effective antituberculosis activity. As shown in Fig. 6, a daily dose of RFP-PLGA for 7 days decreased the bacterial burden to approximately a hundredth compared with that in the group without any treatment. However, insufflation of RFP-PLGA every other day with a total of four doses showed approximately a tenth reduction in bacterial burden compared with that in the untreated group. These results indicate that the daily administration of RFP-PLGA is required for efficient antituberculosis activity because of the faster release and clearance of RFP from RFP-PLGA in the lung, even when RFP is encapsulated in PLGA microspheres intended for sustained release.

#### Device for Insufflation

**Fig. 7:**
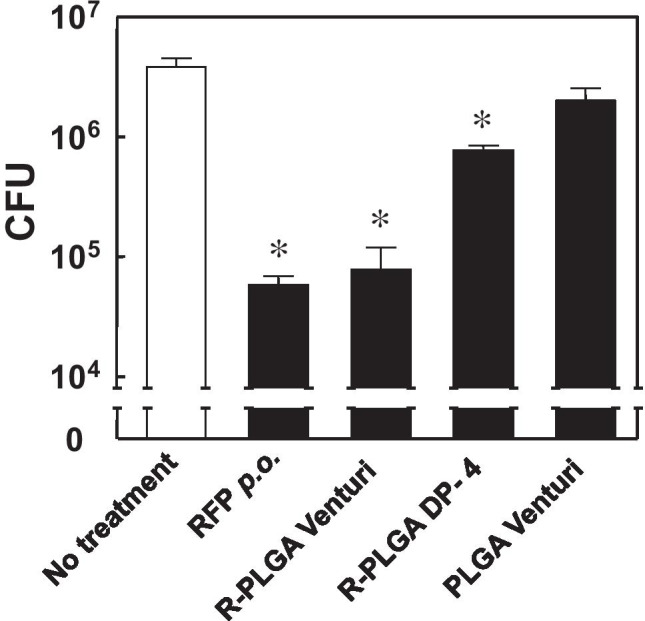
Comparison of the antituberculosis activity of RFP-PLGA insufflated by the Venturi-effect device with that by DP-4. A total of 5 mg of RFP-PLGA (2.8 mg RFP/kg) or blank PLGA was insufflated by the Venturi-effect device or DP-4 to the BCG-infected rats once a day for 7 days, and then CFU in the lung was determined. The effect of oral administration of RFP at 10 mg/kg for 7 days was also examined. The data are expressed as mean ± S.D. (*n* = 3–4). The asterisk indicates statistically significant difference at *p* < 0.05 as determined by Bonferroni’s test.

We then compared the efficiency of the Venturi-effect device with that of DP-4 by determining bacterial burdens after the insufflation of RFP-PLGA containing 2.8 mg/kg RFP (30% of clinical dose) to the rat lung. As shown in Fig. 7, the CFU of the rats treated with DP-4 decreased to 20% of the rats without any treatment. Insufflation with the Venturi-effect device decreased the CFU value to 2% of that without any treatment, and it was almost equivalent to the CFU value attained by the conventional oral administration of RFP, although the dose was approximately 70% less than that of oral administration.

#### Combination of Oral and Pulmonary Doses

**Fig. 8:**
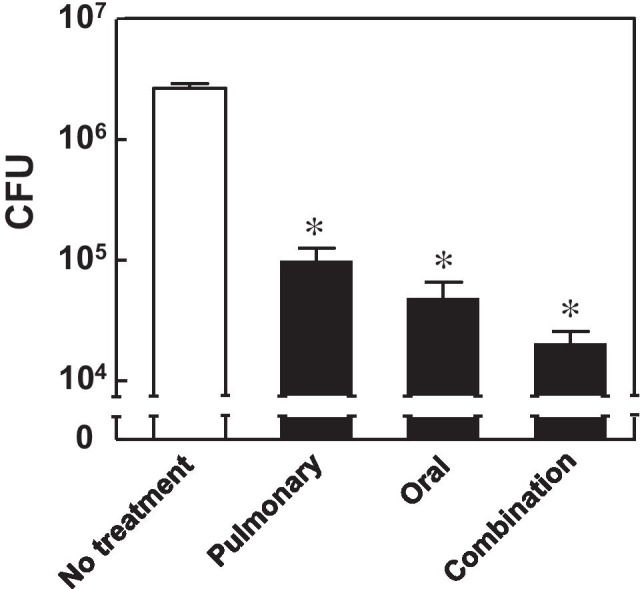
Effect of the combination of RFP delivery by insufflation and oral administration on the antituberculosis activity in the rat lung infected with BCG. RFP-PLGA at 5 mg containing 2.8 mg RFP/kg and RFP at 6 mg/kg was administered together to BCG-infected rats through pulmonary and oral routes, respectively. Individual dose administration of pulmonary and oral routes was performed with RFP-PLGA at 2.8 mg RFP/kg and RFP at 10 mg/kg, respectively. The data are expressed as mean ± S.D. (*n* = 4–5). The asterisk indicates statistically significant difference at *p* < 0.05 as determined by Bonferroni’s test.

Pulmonary insufflation of RFP-PLGA successfully reduced the bacterial burden in the lungs, although this effect seemed insufficient to replace the oral administration of RFP. As mycobacteria are typically found in the granuloma and induce angiogenesis to obtain oxygen and nutrients (23–26), a certain amount of RFP administered orally should reach the pathogen through the systemic blood circulation. We assumed that combined administration routes (i.e., pulmonary and oral pathways) allowed RFP to be efficiently delivered to the infected sites both close to and far (i.e., alveolar side) from blood vessels in the lung. Similar to the above results, pulmonary insufflation of RFP (2.8 mg/kg) in RFP-PLGA showed antituberculosis activity almost equivalent to that by the oral administration of RFP at 10 mg/kg, with approximately 98% reduction in bacterial burden compared with that in the infected rats without any treatment (Fig. 8). A combinational dose of 2.8 mg/kg RFP by pulmonary insufflation and 6 mg/kg RFP by oral administration was more effective in reducing bacterial burden than their individual administration. More than 99% of mycobacteria were killed by daily combined administration for a week.

## Discussion

In this study, we developed a novel insufflator based on the Venturi-effect that enables cohesive dry powders to be aerosolized with a gentle airflow. As slow flow ejection helps the aerosols to decrease their momentums, contrary to DP-4, which expels drugs in a bolus manner, the Venturi-effect device circumvents the entrapment of dry powders in the trachea and bronchi. In addition, an open flow duct of the Venturi-effect device allows the supply of dry powder in a stepwise manner and prevents clogging of the duct with agglomerated powders. Namely, our novel device was able to insufflate a large amount of dry powder to more than 5 mg per single administration procedure.

Previous studies have shown that the inhalation of RFP-PLGA powders was inferior to oral treatment, but partially effective on the mycobacteria residing in the lung (3, 6, 17). It is speculated that the delivery of dry powder loaded with anti-mycobacterial agents to specific areas, which are difficult to reach due to an aerodynamic structure, would optimize the inhalation treatment for tuberculosis. In the present study, a uniform distribution of the dry powder in the lung was achieved by utilizing the Venturi-effect-based insufflator, which resulted in an effective reduction in the mycobacteria residing in the lung, for the first time.

To achieve a uniform distribution of inhalable dry powders in the lung, there are various hurdles even when using the Venturi-effect-based insufflator. The most important point is that dry powders can be aerosolized easily with a low air-flow rate and low pressure that are in the normal range for inhalation. As inhalable dry powders often display coagulating properties caused by cohesiveness and adhesiveness (27, 28), the development of formulations producing fine and dispersed powders is necessary (29).

From the lung structure point of view, the distribution of dry powder depends on the bifurcation number of the bronchi(ole) in the lobe, as more entrapment of dry powders occurs in the deeper lung layers due to complicated routes and turbulence. In addition, the air remaining between the mouth and the lung prevents the inhaled aerosol from reaching the periphery of the lung. One time aerosolization driven by deep inhalation tends to generate fast air flow, which increases the rate of entrapment by bronchi(ole), and this inhalation mode is insufficient to generate homogeneous aerosols, which are delivered to the space occupied with residual air. Utilizing an air ventilator is one of the solutions to deliver dry powders to such spaces because of repeated inhalation, enabling the mixing of aerosols with the residual air. It is known that a high frequency of ventilation enhances the mixing of freshly introduced air with residual air (30). Development of dry powder feeding for high-frequency ventilation would be an ideal solution to achieve uniform distribution in the lung, although dry powders administered but exhaled require management (e.g., development of circuit airways where exhaled dry powders are returned and then re-administered by ventilation).

It is interesting to note that combinational dosing via the pulmonary and oral routes showed a more potent mycobactericidal effect than individual administration. *M. tuberculosis* recruits leukocytes to the infection site to develop the granuloma, which prevents the penetration of antimycobacterial agents (31). Therefore, it would be difficult for inhaled RFP-PLGA microspheres to reach the mycobacterial lesion because they are intended to target infected macrophages assumingly present at the core of the granuloma through endocytic uptake. However, in this study, it is interesting to note that a uniform delivery of RFP-PLGA microspheres displayed potent efficacy against mycobacteria. The following are some reasons that RFP-PLGA microspheres exhibited a significant antimycobacterial activity: 1) infection with BCG enhanced the endocytic uptake activity of macrophages, which are a potential target in our strategy (32); 2) penetration of RFP released from microspheres into the granuloma occurred effectively because locally accumulated RFP in the lung generated a higher gradient of concentration than that in the vein; and 3) the granulomas were immature because an attenuated BCG strain was utilized for the infection model and treatment started a day after inoculation. On the contrary, *M. tuberculosis* enhances angiogenesis to grow and spread in the infection sites efficiently (33), and expands the residual site from the alveolar region to the inner areas, such as interstitial and intravascular tissues in which macrophages with the potential to be infected with *M. tuberculosis* exist (34–37). The transition of the mycobacteria-residing location provides opportunities for antimycobacterial agents to attack through the bloodstream. Indeed, in the initial phase of treatment, the reduction in mycobacterial burden by oral dose clearly proceeds (38). However, in the later phase of infection, oral treatment has to be continued for a long duration, usually approximately 4 months. This indicates that it is difficult to target many mycobacterial cells with the antimycobacterial agents via the bloodstream, potentially due to the presence of granulomas (39). The mechanisms by which pulmonary administration is effective for tuberculosis treatment are still unclear; however, pulmonary and oral administrations would increase drug concentration in the lungs, such as alveoli and interstitial tissues. Thus, combinational administration via different routes is considered a potential therapeutic approach for tuberculosis, as proposed elsewhere (40).

As described above, pulmonary administration of inhalable dry powders with a Venturi-effect device and a ventilator demonstrated high efficacy on a pulmonary disease. Most important point to implement Venturi-effect device is that this approach efficiently delivers dry powders to the lungs and requires much less amount of formulations to be subjected for efficacy and pharmacokinetic study compared to conventional method by nose-only inhalation. Therefore, even in early phase of research, it will be possible to conduct animal studies. In contrast, complex handling of the insufflator under ABSL3 conditions would be challenging. For human use, intubation of the device into the trachea should be limited to treatment of severe diseases. For future studies, designing more convenient type of device (e.g. mouthpiece adapter type administrator) is necessary for developing a widely applicable dry powder administrator. In addition to that, anesthesia is imperative for an administration method proposed here differently from nose-only inhalation. Impact of anesthesia, that usually causes hypotension, respiratory depression, etc., on the drug disposition and the antituberculosis activity should be taken into consideration. Use of the ventilator should minimize an impact of respiratory depression caused by anesthesia.

## Conclusions

Our novel Venturi-effect device 1) aerosolized inhalable antituberculosis dry powders efficiently with a moderate airflow, 2) achieved uniform pulmonary distribution, and 3) aided inhalable RFP-PLGA to exert antituberculosis activity on lung-residing mycobacteria. To achieve effective treatment of pulmonary tuberculosis by inhalation, devices that enable the delivery of inhalable dry powders into the lung in a homogeneous manner are essential. The antituberculosis activity was further potentiated in combination with an oral dose of RFP.
